# Wolfe’s Schiedam Schnapps

**Published:** 1854-01

**Authors:** 


*Wolfe's Schiedam Schnapps.*—We have received a pamphlet containing a laudation of this form of the "Batavian Elixir," with a number of certificates from physicians and the press, as to its purity and medical virtues, which is perfectly overwhelmning. We have entire confidence in the sincerity and good faith of these endorsers; they were undoubtedly good judges of the article.

As the Schiedam Schnapps are nothing less than very good *gin*, we can understand their popularity. A lively friend informs us, that their free use during the excessively warm weather of summer, prevents profuse and unpleasant perspiration by increasing the secretions of the kidneys, and obviates some of the dangers of cholera, by maintaining the activity of those organs—a long-sought desideratum. We shall wait for further evidence before we express an opinion. S. B. H.

